# Unusual Nitrogenous Phenalenone Derivatives from the Marine-Derived Fungus *Coniothyrium cereale*

**DOI:** 10.3390/molecules21020178

**Published:** 2016-02-01

**Authors:** Mahmoud Fahmi Elsebai, Hazem A. Ghabbour, Mohamed Mehiri

**Affiliations:** 1 Department of Pharmacognosy, Faculty of Pharmacy, Mansoura University, Mansoura 35516, Egypt; 2 Department of Pharmaceutical Chemistry, College of Pharmacy, King Saud University, Riyadh 11451, Saudi Arabia; ghabbourh@yahoo.com; 3 Department of Medicinal Chemistry, Faculty of Pharmacy, Mansoura University, Mansoura 35516, Egypt; 4 Institut de Chimie de Nice (ICN), Faculté des Sciences, Université Nice Sophia Antipolis, UMR CNRS 7272, Parc Valrose, Nice 06108 Cedex 02, France; mehiri@unice.fr

**Keywords:** marine natural products, *Coniothyrium cereale*, endophytic fungal phenalenones, conio-azasterol, *S*-dehydroazasirosterol, molecular docking

## Abstract

The new phenalenone metabolites **1**, **2**, **4**, and **6** were isolated from the marine-derived endophytic fungus *Coniothyrium cereale*, in addition to the ergostane-type sterol (**3**) and entatrovenetinone (**5**). Compounds **1** and **2** represent two unusual nitrogen-containing compounds, which are composed of a sterol portion condensed via two bonds to phenalenone derivatives. Compound **6**, which contains unprecedented imine functionality between two carbonyl groups to form a oxepane -imine-dione ring, exhibited a moderate cytotoxicity against K562, U266, and SKM1 cancer cell lines. Moreover, molecular docking studies were done on estrogen receptor α-ligand binding domain (ERα-LBD) to compounds **1** and **2** to correlate with binding energies and affinities calculated from molecular docking to the anti-proliferative activity.

## 1. Introduction

**Figure 1:**
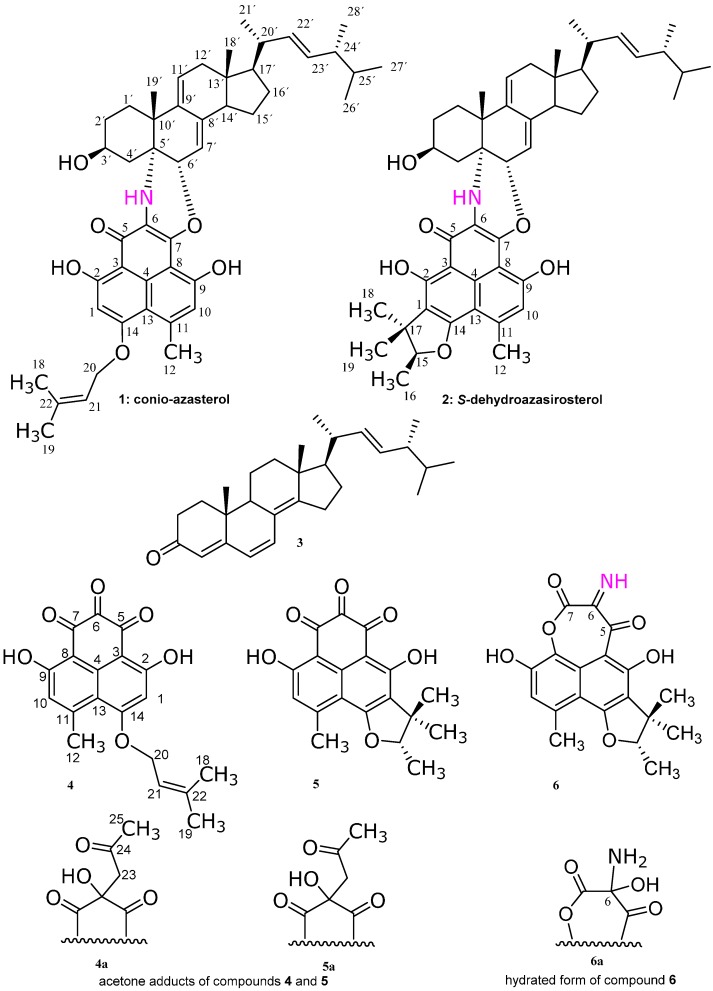
Chemical structures of the secondary metabolites **1**–**6** isolated from *Coniothyrium cereale*.

Phenalenone derivatives have been reported both from higher plants and microbial sources. They possess significant biological and chemical importance [1]. Marine fungi represent a quite diverse group and an excellent source of natural products where they represent a huge potential for new chemical metabolites. The production of metabolites from fungi is a rapidly growing field as can be observed from the increased number of reviews concerned with this topic and the increased number of new natural products which have become known over the past years [2]. Studies of the endophytic marine fungi indicate that they are prolific producers of unique natural products, which render them as highly useful in the drug discovery process and indicating that much of their hidden potential still needs to be uncovered [3]. The marine-derived endophytic fungus *Coniothyrium cereale* was isolated from the Baltic Sea algae *Enteromorpha* sp. Cultivation of the fungus *C. cereale* in media supplemented with sea salt led to structurally unusual nitrogenous phenalenone derivatives **1**, **2**, and **6**. In this paper, we report the unusual heterodimer phenalenone metabolites **1** and **2** which are constructed from sterol and polyketidic phenalenone derivatives (Figure 1). We report compound **6** here also which has unusual imine functionality between two carbonyl groups. Moreover, we report the ubiquitous fungal ergostane-type sterol (22*E*,24*R*)-Ergosta-4,6,8(14)22-tetraen-3-one (**3**), and entatrovenetinone (**5**) from the same fungus. As a preliminary test for biological activity, products were screened for their antimicrobial and cytotoxic activities and the results were correlated with molecular docking studies on estrogen receptor α-ligand binding domain (ERα-LBD).

## 2. Results and Discussion

Subfraction 11 of the ethyl acetate extract of the mycelium of *C. cereale* was subjected to RP-HPLC separation to give the yellowish brown compounds **1** and **2**. At first glance, from their ^1^H- and ^13^C-NMR spectra, they were suspected to be a mixture of a sterol and a phenalenone like compounds, though the HPLC chromatogram showed only one peak for each compound. Further elucidation of the mass gave a signal at 733 Da correspondent to a molecular formula C_47_H_59_NO_6_, suggesting that compounds **1** and **2** are adducts of a sterol and phenalenone derivatives. Further investigation of the 1D and 2D NMR spectra confirmed the adduct formation as follows.

Compound **1** showed a molecular formula of C_47_H_59_NO_6_ on the basis of the number of signals in both ^1^H- and ^13^C-NMR spectra and accurate mass measurement (HRESIMS: *m*/*z* found = 756.4235 [M + Na]^+^, Figure S2). The ^1^H-NMR spectrum (Figure S1) of the phenalenone portion of **1** gave rise to signals for two exchangeable phenolic hydrogens, one is strongly chelated (δ*_H_* 16.91 for 2-OH) with a carbonyl group (IR 1711/3354 cm^−1^), and the second is weakly chelated (δ*_H_* 9.69 for 9-OH), in addition to a characteristic NH resonance at δ*_H_* 3.93 which has no correlation in the HSQC (Figures S3–S6).

**Figure 2:**
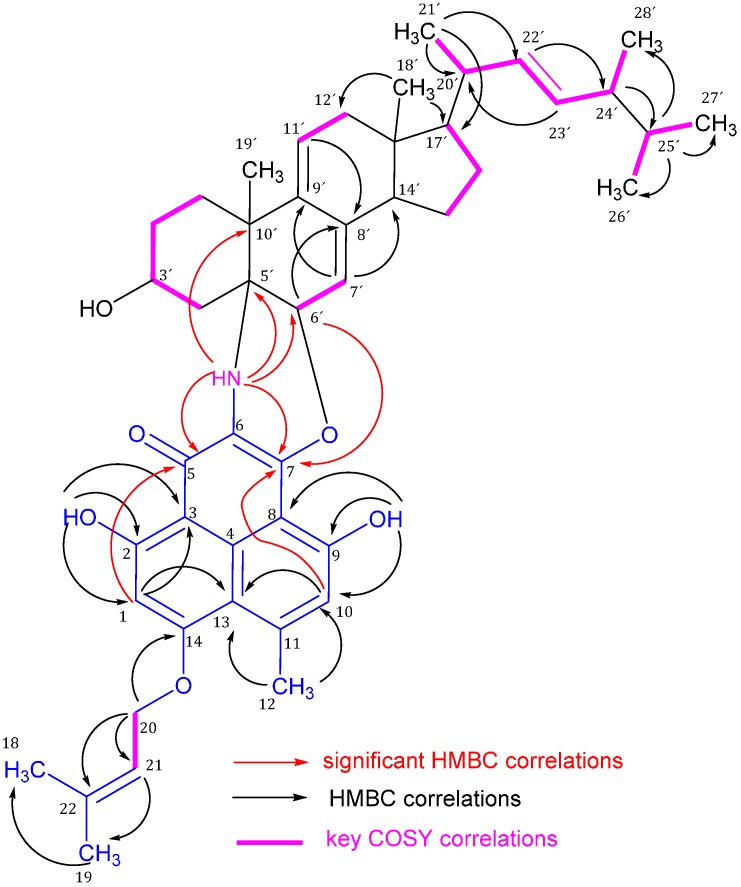
Significant ^1^H-^13^C HMBC correlations (arrows, proton to carbon) and ^1^H-^1^H COSY (bold lines) of compound **1**.

The ^1^H- and ^13^C-NMR spectra showed an aromatic methyl (δ*_H/C_* 2.78/25.9 for CH_3_-12) and two aromatic protons (δ*_H_* 6.38 and 6.81 for H-1 and H-10, respectively). A UV maximum at 395 nm clearly evidenced that compound **1** has an extended aromatic system. Further two ^1^H-NMR singlet resonance signals arose from aromatic protons (δ*_H_* 6.38 for H-1 and δ*_H_* 6.81 for H-10). These aromatic protons (H-1 and H-10), each had a distinctive set of correlations in the ^1^H-^13^C HMBC spectrum suggesting that each of these protons is attached to a different benzene ring. In the ^1^H-^13^C HMBC spectrum, H-1 showed cross peak correlations with C-2, C-3, C-5, C-13, and C-14, whereas H-10 had correlations with C-7, C-8, C-9, C-12, and C-13. H_3_-12 had heteronuclear couplings to C-10, C-11, and C-13. 2-OH showed cross peak correlations with C-1, C-2, and C-3; and 9-OH with C-8, C-9, and C-10. This pattern of heteronuclear correlations, together with the ^1^H- and ^13^C-NMR data indicated for a naphthalene-type compound of two connected penta-substituted benzene rings, substituted at C-2 and C-9 with phenolic groups and at C-11 with a methyl group. The presence of the 3-methyl-2-butenyl group in **1** was proven as follows: the ^1^H- and ^13^C-NMR spectrum contained two singlet resonances at δ*_H/C_* 1.81/25.8 for CH_3_-18 and δ*_H/C_* 1.76/18.3 for CH_3_-19 due to a geminal dimethyl group attached to an olefinic carbon. This was corroborated by the HMBC cross peak correlations between H_3_-18 and H_3_-19 and C-22. The downfield shifted doublet at δ*_H/C_* 4.69/66.0 is assigned for the methylene protons CH_2_-20 which is attached to oxygen and the methine triplet at δ*_H/C_* 5.56/118.4 is assigned for CH-21. The ^1^H-^1^H COSY spectrum showed cross peak correlations for a ^1^H-^1^H-spin system ranging from both terminal methyl protons via H-21 to H_2_-20. The prenylation occurred at the oxygenated carbon C-14 due to the HMBC correlation of H_2_-20 to C-14 as depicted in Figure 2. These chemical shifts and correlations are similar to those of the compound coniosclerodin [4].

The sterol portion of **1** gave rise to ^1^H- and ^13^C-NMR signals (Table 1) very similar to those of a sterol compound related to an ergosterol. Thus, the methyl groups of the sterol portion produced singlets at δ*_H/C_* 0.50/11.6 and 1.23/23.6 for the angular tertiary CH_3_-18′ and 19′, respectively, and four doublets at δ*_H/C_* 0.96/20.7, 0.76/19.6, 0.78/19.9, and 0.85/17.6 for the secondary methyl groups CH_3_-21′, 26′, 27′, and 28′, respectively, of the sterol side chain. The two alkenic CH groups of the side chain gave rise to double doublets at δ*_H/C_* 5.07/135.2 and 5.17/132.2 for CH-22′ and 23′, respectively. The ^1^H-^1^H COSY and ^1^H-^13^C HMBC correlations (Figures S9 and S10) resulted in a sterol side chain of nine carbons with one olefinic double bond to give an ergostene side chain (Figure 2). Further two olefinic CH groups resonating at δ*_H/C_* 5.00/116.9 and 5.68/124.9 are assigned to CH-7′ and 11′, respectively, to form an exocyclic diene system with the quaternary carbons C-8′ and C-9′ due to HMBC correlations as illustrated in Figure 2. These structural features were confirmed by the HMBC correlations as illustrated in Table 1 and Figure 2. The methine multiplet at δ*_H/C_* 4.10/66.7 has the expected complexity of steroidal 3-carbinol hydrogen which is characteristic for 3β-hydroxysterols. The steroidal nucleus is attached to the phenalenone part through two bonds. The first bond is from C-5′ to C-6 through a NH bridge and this is confirmed by the significant HMBC correlations of NH to C-5, 7, 4′, 5′, 6′, and 10′. The second bond is from CH-6′ to C-7 via oxygen due to the HMBC correlation of H-6′ to C-7 (Figure 2 and Table 2).

**Table 1:** NMR (CDCl_3_) spectroscopic data for conio-azasterol (**1**).

No.	Mult.	δ*_C_* (ppm)	δ*_H_* (ppm), (mult, *J* in Hz)	No.	Mult.	δ*_C_* (ppm)	δ*_H_* (ppm), (mult, *J* in Hz)
1	CH	97.4	6.38, s	4′	CH_2_	37.0	a: 1.52, m b: 2.12, m
2	C	173.9		5′	C	55.5	
3	C	104.4		6′	CH	75.8	4.85, brs
4	C	120.6		7′	CH	116.9	5.00, brs
5	C	174.4		8′	C	139.0	
6	C	124.2		9′	C	140.6	
7	C	141.5		10′	C	40.2	
8	C	104.2		11′	CH	124.9	5.68, brd (5.5)
9	C	157.9		12′	CH_2_	41.9	a: 2.12, m b: 2.30, m
10	CH	117.5	6.81, s	13′	C	42.4	
11	C	143.0		14′	CH	50.9	2.07, m
12	CH_3_	25.9	2.78, s	15′	CH_2_	22.9	1.53, m
13	C	112.5		16′	CH_2_	28.6	1.60, m
14	C	167.3		17′	CH	55.7	1.20, m
18	CH_3_	18.3	1.76, s	18′	CH_3_	11.6	0.50, s
19	CH_3_	25.8	1.81, s	19′	CH_3_	23.6	1.23, s
20	CH_2_	66.0	4.69, d (6.2)	20′	CH	40.3	1.95, m
21	CH	118.4	5.56, brt (6.2)	21′	CH_3_	20.7	0.96, d (6.3)
22	C	139.0		22′	CH	135.2	5.07, dd (7.7, 15.0)
2-OH			16.91, s	23′	CH	132.2	5.17, dd (7.3, 15.0)
9-OH			9.69, s	24′	CH	42.8	1.78, m
NH			3.93, s	25′	CH	33.0	1.40, m
1′	CH_2_	29.7	1.98, m	26′	CH_3_	19.6	0.76, d (6.3)
2′	CH_2_	30.2	1.66, m	27′	CH_3_	19.9	0.78, d (6.3)
3′	CH	66.7	4.10, m	28′	CH_3_	17.6	0.85, d (6.3)

**Table 2:** NMR (CDCl_3_) spectroscopic data for *S*-dehydroazasirosterol (**2**).

No.	Mult.	δ*_C_* (ppm)	δ*_H_* (ppm), (mult, J in Hz)	No.	Mult.	δ*_C_* (ppm)	δ*_H_* (ppm), (mult, J in Hz)
1	C	118.2		5′	C	55.6	
2	C	169.9		6′	C	75.6	4.85, brs
3	C	105.4		7′	CH	116.9	5.00, brs
4	C	120.9		8′	C	140.5	
5	C	174.8		9′	C	139.0	
6	C	124.1		10′	C	40.1	
7	C	140.6		11′	CH	124.8	5.68 d (5.49)
8	C	104.1		12′	CH_2_	41.9	a: 2.13, m
9	C	158.4					b: 2.32, m
10	CH	116.4	6.82, s	13′	C	42.3	
11	C	142.3		14′	CH	50.9	2.10, m
12	CH_3_	23.6	2.80, s	15′	CH_2_	22.7	1.53, m
13	C	108.7		16′	CH_2_	28.5	1.64, m
14	C	166.2		17′	CH	55.7	1.23, m
15	CH	91.2	4.67, q (6.6)	18′	CH_3_	11.6	0.51, s
16	CH_3_	14.6	1.46, d (6.6)	19′	CH_3_	23.5	1.25, s
17	C	43.2		20′	CH	40.3	1.98
18	CH_3_	25.7	1.54, s	21′	CH_3_	20.6	0.98, d (6.6)
19	CH	20.7	1.30, s	22′	CH	135.3	5.10, dd (7.7, 15.0)
2-OH			16.84, s	23′	CH	132.2	5.18, dd (7.0, 15.0)
9-OH			9.76, s	24′	CH	42.8	1.82, m
NH			3.98, s	25′	CH	33.0	1.43, m
1′	CH_2_	29.4	2.01, m	26′	CH_3_	19.6	0.78, d (6.3)
2′	CH_2_	30.3	1.68, m	27′	CH_3_	19.9	0.80, d (6.3)
3′	CH	66.8	4.11, m	28′	CH_3_	17.6	0.88, d (6.3)
4′	CH_2_	37.0	a: 1.54, m				
			b: 2.12, m				

Compound **2** is structurally similar to compound **1**, except that the prenyl moiety is attached to the phenalenone part as a trimethyl dihydrofuran ring (Figure 1) as follows. HMBC correlations (Table 2) allowed connecting the C-15 to C-19 part of the molecule, which is an oxygenated hemiterpene unit. C-17 of this partial structure was attached to C-1 of the aromatic moiety due to heteronuclear long range couplings of H_3_-18 and H_2_-19 to C-1. Ring closure to a dihydrofuran ring occurred via the oxygen atom at C-15 to C-14 of the aromatic nucleus. The complete structural identification of **2** was done by extensive spectroscopic data (Table 1 and Table 2). Compound **2** was isolated in this work as an epimeric pure form for the first time (Figure S7), and it was reported before as an epimeric mixture at C-15 under the name dehydroazasirosterol [5]. Therefore, we gave the name conio-azasterol and *S*-dehydroazasirosterol for compounds **1** and **2**, respectively.

The LC/MS, ^1^H- and ^13^C-NMR spectra (Figures S13 and S14) of compound **3** revealed an ergostane-type compound (ergosteroid) which has the same structure as (22*E*,24*R*)-ergosta-4,6,8(14)-22-tetraen-3-one [6] (Figure 1).

The structures of compounds **4** and **5** were established based on extensive spectroscopic measurements as in Table 3 and Table 4, respectively. Compound **4** could not be isolated in its genuine triketone form but instead as an acetone adduct (**4a**) on C-6 (Figure 1, Figures S15 and S16, and Table 3). The position of acetonyl moiety of the acetone adduct was confirmed by the HMBC correlation of H_2_-23 to C-6. A pair of enantiomers (1:1 mixture) at C-6 was found which was confirmed by the absence of CD Cotton effects and zero optical rotation for **4a**. The acetone adduct of compound **4** (**4a**) is reported under the name rousselianone A′ [7]. Also compound **5** could not be isolated in its genuine triketone form but instead as an acetone adduct (**5a**) on C-6 (Figure 1). The position of acetonyl moiety is confirmed by the HMBC correlation of H_2_-23 to C-6. A pair of epimers (1:1 epimeric mixture) at C-6 was found which was confirmed by the presence of two sets of resonances for **5a** in the ^1^H- and ^13^C-NMR spectra (Table 4, Figures S17 and S18). The complete spectroscopic data of **5a** is listed in Table 4 for the first time. Compound **5** (entatrovenetinone) was formerly separated from *Gremmeniella abietina* [8]. The genuine triketone compounds (**4** and **5**) were recognized *via* LC/MS (*m/z* 340) and HRESIMS at 341.1037 [M + 1]^+^, calc 341.1025 [M + 1]^+^, using additionally prepared extract prepared under nitrogen atmosphere and without subjecting the extract to acetone during the extraction process (Figure S20).

**Table 3:** NMR (CDCl_3_) spectroscopic data for 4a.

No.	δ*_C_* (ppm)	Multi.	δ*_H_* (ppm), (mult, J in Hz)	^1^H-^1^H COSY	^1^H-^13^C HMBC	^1^H-^1^H NOESY
1	97.1	CH	6.37, s		2, 3, 13, 14	20
2	168.8	C				
3	101.8	C				
4	137.1	C				
5	197.1	C				
6	77.7	C				
7	199.2	C				
8	105.5	C				
9	165.6	C				
10	119.1	CH	6.77, s	12	8, 9, 13	12
11	150.0	C				
12	26.6	CH_3_	2.78, s	10	10, 11, 13	10
13	113.7	C				
14	166.5	C				
18	18.3	CH_3_	1.78, s	20, 21	19, 21, 22	20
19	25.8	CH_3_	1.83, s	20, 21	18, 21, 22	21
20	66.3	CH_2_	4.68, d, 6.6	18, 19, 21	14, 21, 22	1, 18
21	117.7	CH	5.54, br t, 6.6	18, 19, 20		19
22	139.9	C				
23	52.0	CH_2_	3.25, s		5, 6, 7, 24	25
24	205.6	C			23, 24	
25	31.1	CH_3_	2.19, s			23
2-OH			13.24, s		1, 2, 3	
9-OH			12.67, s		8, 9, 10	

**Table 4:** NMR (CDCl_3_) spectroscopic data for 5a.

No.	δ*_C_* (ppm)	Mult.	δ*_H_* (ppm), (mult, J in Hz)	^1^H-^1^H COSY	^1^H-^13^C HMBC	^1^H-^1^H NOESY
1	118.4/118.5	C				
2	165.5/165.4	C				
3	102.5	C				
4	137.4	C				
5	196.9/196.8	C				
6	77.1/77.3	C				
7	199.2	C				
8	105.4	C				
9	166.2/166.1	C				
10	117.9	CH	6.74, br s	12	8, 9, 12, 13	12
11	149.3	C				
12	24.3	CH_3_	2.76, s	10	10, 11, 13	10
13	109.7	C				
14	166.1	C				
15	91.7/91.6	CH	4.64, q (6.6)	16	17, 18, 19	16, 18
16	14.7	CH_3_	1.46, d (6.6)	15	15, 17	15, 19
17	43.3	C				
18	25.7	CH_3_	1.51/1.52, s		1, 17, 19	15
19	20.6	CH_3_	1.30/1.27, s		1, 15, 17, 18	16
23	51.8/52.1	CH_2_	3.31/3.27, s		5, 6, 7, 24	
24	206.1/205.9	C				
25	31.1/31.0	CH_3_	2.20, s		24	
2-OH			13.34/13.29, s		1, 2, 3, 5	
9-OH			12.81/12.78, s		8, 9, 10	

Compound **6a** was elucidated based on extensive spectroscopic measurements (Figures S21–S25). Compound **6** has the same structural features as in compound **5**, except that there is unique imine moiety at C-6 and lactone oxygen between C-8 and C-7. Compound **6** could not be isolated in the imine form, but in its hydrated one (Figure 1). The latter was suspected due to the presence of epimeric mixture (1:1 ratio) at C-6 corroborated from the two sets of resonances in ^1^H- and ^13^C-NMR spectra (Figures S21 and S22). The attachment of oxygen to C-8 was proven from its chemical shift at 130.2 ppm in the ^13^C-NMR spectrum (Table 5). The latter is similar to the chemical shifts in the lactone and the ketolactone compounds reported by Elsebai *et al.* [4]. The presence of a nitrogen atom was suggested by the odd numbered molecular mass at 373 Da (HRESIMS *m*/*z*, found = 396.1057 [M + Na]^+^). The position of the imine at C-6 is supported by the structure of compounds **1** and **2** where the nitrogen atom is at the same position which is the mid carbonyl group. This is also chemically the most plausible position because the mid carbonyl group of a triketone system is the most active one for a transamination reaction. In addition, the respective carbon chemical shifts were in agreement with the values calculated by the ACD NMR predictor software^®^ (ACD laboratories) for **6a**. We report here the complete spectroscopic data (Table 5) for the hydrated form of compound **6** (**6a**).

**Table 5:** NMR (CD_3_COCD_3_) spectroscopic data for **6a**.

No.	δ*_C_* (ppm)	Mult.	δ*_H_* (ppm), (mult, J in Hz)	^1^H-^13^C HMBC	^1^H-^1^H NOESY
1	117.9	C			
2	169.2	C			
3	94.9	C			
4	125.8	C			
5	189.1/189.2	C			
6	119.1	C			
7	157.1	C			
8	130.2	C			
9	146.7	C			
10	118.5	CH	6.81, s	8, 9, 12, 13	
11	132.0	C			
12	22.1	CH_3_	2.66, s	10, 11, 13, 14	
13	109.8	C			
14	167.2	C			
15	92.06/92.13	CH	4.69, q (6.6)	14, 17, 18, 19	16, 18
16	14.8/14.7	CH_3_	1.51, d (6.6)	15, 17	19
17	43.9/43.8	C			
18	25.8/26.0	CH_3_	1.53, s	1, 15, 17, 19	15
19	20.9/21.0	CH_3_	1.30, s	1, 15, 17, 18	16
2-OH			12.99		
9-OH			11.98		

For the stereochemistry of compounds **1**–**6**, compound **2** has the all stereogenic centers as in compounds **1**–**6**. Due to the similarity in NMR chemical shifts for both compounds **1** and **2** and their identical biogenetic origin, they should have the same stereochemistry of the sterol portion; and likewise for compounds **5** and **6**, which have the phenalenone stereogenic centre at C-15 as in compound **2**. Therefore, the stereochemistry of compound **2** is discussed in details as follows.

For the sterol portion, both the type and orientation of substituents in the steroid nucleus affect their ^13^C-NMR chemical shifts [9]. Consequently, the orientation of 3′-OH is β due to the ^13^C chemical shift of C-3′ at δ*_C_* 66.7 for compound **1** and δ_C_ 66.8 for compound **2** (the α-carbinol C-3′ has chemical shift around δ*_C_* 71.0 [10]. The NH bridge is α-oriented due to the NOESY correlation of NH to H-3′ (Figures S11 and S12) which is further confirmed by enhancing the H-3′ resonance signal upon irradiation of NH resonance using 1D-NOE measurement. Also, the absence of a NOESY correlation between CH_3_-19′ and NH further confirmed the α-orientation of the amine bond. A NOESY correlation between H_3_-19′ and H-6′ indicated the oxygen bridge between C-7 and C-6′ to be α-oriented and in turn the whole phenalenone nucleus due to the planarity of the aromatic system. The phenalenone nucleus is therefore perpendicular to the flattish sterol part due to the aforementioned NOESY correlations and the *trans* fusion of the steroid rings A/B and C/D in addition to the planar arrangements of the middle atoms of the sterol nucleus with the C-C double bonds. H_3_-18′ has no NOESY correlation to H-14′ confirming the *trans* fusion of the steroid ring C/D and this is matching with the *transoid* nature of most natural steroids. H_3_-19′ has a NOESY correlation to H_3_-18′. The β-orientation of the CH_3_-19′ group was further confirmed by its enhancement upon irradiation at the H_3_-18′ using a 1D-NOE measurement.

The stereochemistry of the sterol side chain was determined to be as shown in Figure 1. It was determined by comparison of the ^13^C-NMR data of compound **2** with those of the (22*E*,24*R*)-methyl-Δ^22^-sterol side chain of known steroids [11,12]. Wright *et al.*. [12] studied the ^13^C-NMR spectra of diastereomeric C-24 alkyl sterols and they found that the differences in the ^13^C-NMR chemical shifts of side-chain carbons permitted the determination of the absolute configuration at C-24, and stated that the absolute configuration of the sterol side chain can be determined by the ^13^C chemical shifts of the respective carbons. Wright *et al.*. [12] found that specifically the resonance for the C-28′ methyl carbon appears at a characteristic value of 17.6 ± 0.1 ppm in the 24*R* epimer. Interestingly, in compounds **1**–**3**, the ^13^C chemical shifts of C-28′ are the same which is 17.6 ppm, although the main sterol skeletons in compounds **1**–**3** are not similar, indicating the *R* configuration at C-24′ for all of them. This is possible since these chemical shifts are insensitive to structural changes remote from the C-24′ stereogenic centre [12]. Further confirmation of the 24′*R* configuration was described by Goad *et al.*. [13] who recognized that many sterols of plants and microorganisms contain a methyl or ethyl group at C-24′, and both C-24′ diastereoisomers have been found to occur naturally. Goad *et al.*. [13] established that there appears to be some phylogenetic significance to the configuration at C-24′, since, in general, algae and fungi produce sterols with the 24*′R* configuration (24′*S* if a saturated side chain, 24′*R* in the Δ^22^ derivative) whereas sterols in most vascular plants possess the 24′*S* configuration. The presence of NOESY correlation between H_3_-18′/H-20′ and between H-14′/H_3_-21′ indicated that the sterol side chain is β-oriented to the main sterol nucleus and the *transoid* nature of rings C/D (Figure 1). We assume a *S* configuration of the single stereogenic center of the phenalenone nucleus at C-15 for compounds **2**, **5**, and **6** based on the same biogenetic origin as the previously reported compounds from the same fungus [4,14].

All the compounds were evaluated for their antimicrobial and cytotoxic activity. All the compounds showed marginal antibacterial activities in agar diffusion assays against *Staphylococcus aureus*, *Escherichia coli*, *Pseudomonas aeruginosa*, and *Candida albicans*. The cytotoxicity of compounds **1**, **2**, **4a**, **5a**, and **6a** was evaluated against SKM1, U266, and K562 cancer cell lines which are widely used for cytotoxicity assays, and the IC_50_ values in µM (XTT assay). Only compound **6a** exhibited moderate cytotoxic activities with IC_50_ values between 75, 45, and 8.5 µM against SKM1, U266, and K562 cancer cell lines, respectively (*n* = 3).

### Molecular Docking Study

**Figure 3:**
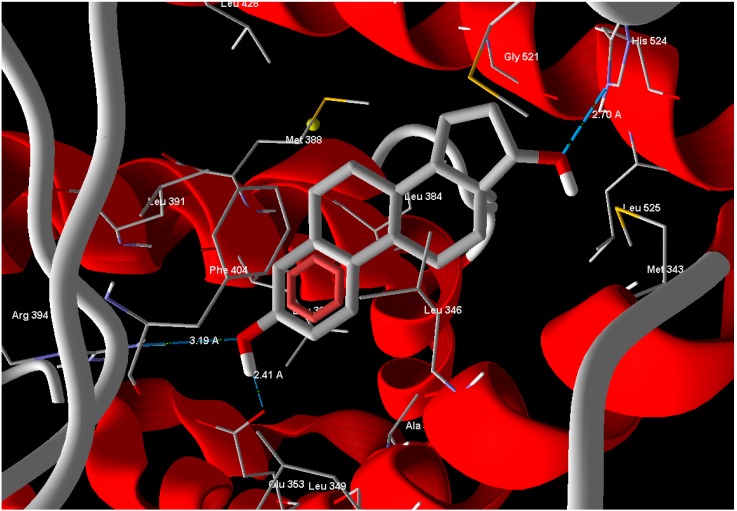
Estrogen showed hydrogen bonds interactions with ERα-LBD active site.

In silico docking is a very famous method employed to investigate molecular association. The knowing of the binding capabilities of the active site residues to specific groups on the agonist or antagonist leads to various strategies for the synthesis of very specific agents with a high probability of biological action. In silico docking experiments were conducted for compounds **1** and **2** against the X-ray crystal structure of b-estradiol-bound ERα receptor ligand binding domain (ERα-LBD, PDB: 1A52) [13], to compare the binding affinity of the tested compounds. Estrogen (standard drug) reveals Mol-Dock score of −105.9 and forms three hydrogen bonds between its phenolic OH moiety and Glu 353 with a bond distance of 2.41 Å and another bond with Arg 394 with bond distance of 3.19 Å and the alcoholic OH form one hydrogen bond with His 524 with bond distance of 2.70 Å (Figure 3). Compounds **1** and **2** exhibited binding scores −77.61 and −18.54, respectively. Compound **1** has relatively good MolDock score and made two hydrogen bonds with same amino acids in the active site of ERα-LBD.

**Figure 4:**
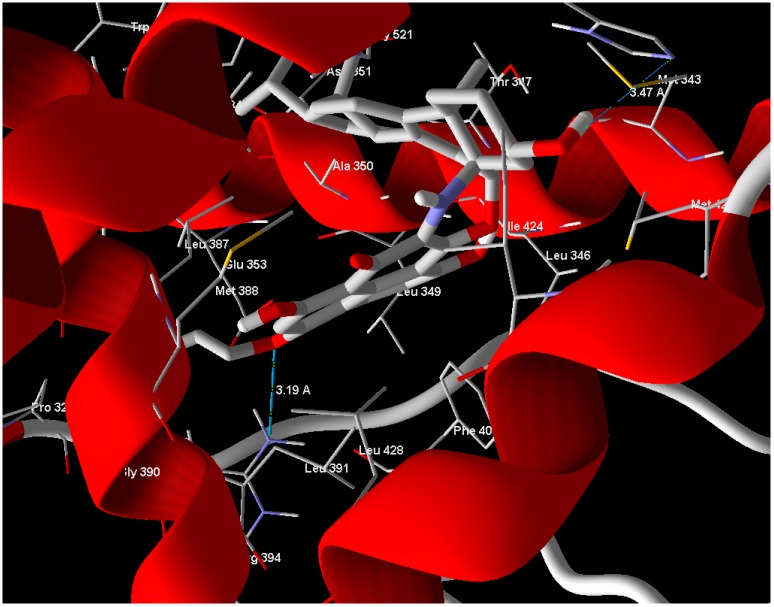
Compound **1** showed hydrogen bonds interaction with ERα-LBD active site.

**Figure 5:**
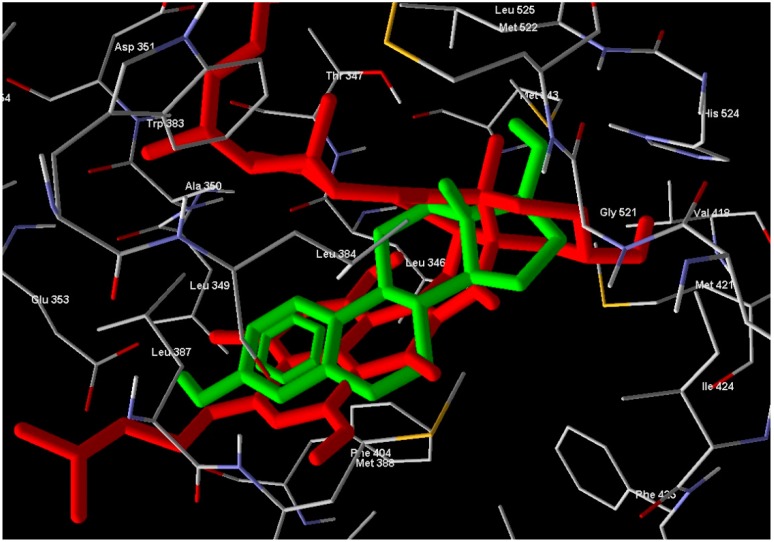
Compound **1** (red molecule) superimposed with standard drug (green molecule) in ERα-LBD active site (Color figure online).

Arg 394 and His 524 with bond lengths 3.19 Å and 3.47 Å, respectively, in addition to hydrophobic interaction with Phe 404 and Trp 383 (Figure 4). The position of compound **1** in the active site overlaps well with Estrogen (standard drug) (Figure 5) and this means a good quality of the docking process. Compound **2** has relatively very low MolDock score and weak interaction with receptor.

## 3. Materials and Methods

### 3.1. General Experimental Procedures

UV and IR spectra were obtained employing Perkin-Elmer Lambda 40 and Perkin-Elmer Spectrum BX instruments (PerkinElmer, Inc., Waltham, USA), respectively. VLC grade (Macherey-Nagel, Polygoprep 60-50C18) was used for vacuum liquid chromatography. All organic solvents were distilled prior to use. HPLC was carried out using a Waters system, controlled by Waters Millenium software, consisting of a 600E pump, a 996 PDA, and a 717 plus autosampler (Waters Corporation, Milford, MA, USA). All NMR spectra were recorded on a Bruker Avance 300 and 500 DRX spectrometers (Bruker Corporation, Rheinstetten, Germany). Spectra were referenced to the residual solvent signals with resonances at δ*_H/C_* 2.04/29.8 (acetone-*d*_6_) and δ*_H/C_* 7.26/77.0 (CDCl_3_). ESI mass spectra were obtained on an Applied Biosystems/MDS Sciex API 2000 MS spectrometer (Applera Corporation and MDS Inc., Foster, CA, USA). HRESIMS were recorded on a Bruker Daltonik micrOTOF-Q Time-of-Flight mass spectrometer with ESI source, and UPLC-Synapt G2 HDMS mass spectrometer (Bruker CorporationCompany, Rheinstetten, Germany).

### 3.2. Origin of the Algal Sample and Isolation of the Fungus

An algal sample *Enteromorpha* sp. was collected from Fehmarn, Baltic Sea. The isolation of the fungus from the host tissues was carried out through single colony isolation method as follows. Algal samples were rinsed three times with sterile H_2_O. After surface sterilization with 70% EtOH for 15 s the algae was rinsed in sterile artificial seawater (ASW). Subsequently, the algae were aseptically cut into small pieces and placed on agar plates containing isolation medium: agar 15 g/L, ASW 800 mL/L, glucose 1 g/L, peptone from soymeal 0.5 g/L, yeast extract 0.1 g/L, benzyl penicillin 250 mg/L, and streptomycin sulfate 250 mg/L. The fungus growing out of the algal tissue was separated on biomalt medium (biomalt 20 g/L, agar 10 g/L, ASW 800 mL/L) until the culture was pure.

### 3.3. Cultivation, Extraction and Isolation of Compounds ***1**–**6***

The fungal strain *Coniothyrium cereale* was cultivated for 40 days on 10 L solid BMS medium (Gesundheitsprodukte GmbH, Kirn, Germany) with agar (15 g/L) at room temperature in 40 Fernbach flasks. Afterwords, the fungal biomass and media were homogenized using an Ultra-Turrax apparatus and extracted with 8 L EtOAc to yield 10.0 g of crude extract. This material was fractionated by RP VLC using a stepwise gradient solvent system of increasing polarity starting from 50% methanol and 50% water to 100% methanol which yielded 12 fractions. RP-HPLC separation of the subfraction 11 (column: Waters Atlantis C_18_, 250 × 4.6 mm, 5 µm; methanol/H_2_O (95:5), flow rate 2 mL/min) afforded compounds **1**–**3**. RP-HPLC separation of the subfraction 6 (column: Waters Atlantis C_18_, 250 × 4.6 mm, 5 µm; acetonitrile/H_2_O (70:30), flow rate 1.5 mL/min) afforded compounds **4**–**6**.

*Conio-azasterol* (**1**): Yellowish brown powder (7.5 mg; 0.7 mg·L^−1^); UV λ_max_ MeOH (log ε): 395 (4.90), 245 (4.11) nm; ${\left[\alpha \right]}_{D}^{24}$ = +90 (C 0.3, CHCl_3_); IR νmax (ATR): 3354, 2919, 2850, 1741, 1711, 1609, 1461, 1371, 1193, 1064, 1032, 977 cm^−1^; ^1^H- and ^13^C-NMR see supp. info. Table 1; (+)-HRESIMS: *m*/*z* found = 756.4235 [M + Na]^+^ and *m*/*z* calcld = 756.4240 [M + Na]^+^.

*S-Dehydroazasirosterol* (**2**): Yellowish brown powder (9.3 mg; 0.9 mg·L^−1^); UV λ_max_ MeOH (log ε): 395 (4.81), 240 (4.50) nm; ${\left[\alpha \right]}_{D}^{24}$ = +35 (C 0.6, CHCl_3_); IR ν_max_ (ATR): 3382, 2919, 2850, 2358, 1737, 1711, 1608, 1461, 1371, 1295, 1193, 1064, 1033, 977, 861 cm^−1^; ^1^H- and ^13^C-NMR see supp. info. Table 2; (+)-HRESIMS: *m*/*z* found = 756.4235 [M + Na]^+^ and *m*/*z* calcld = 756.4240 [M + Na]^+^.

*(22E,24R)-Ergosta-4,6,8(14)22-tetraen-3-one* (**3**): Yellowish brown powder (8.5 mg; 0.8 mg·L^−1^); ^13^C-NMR (75 MHz; CDCl_3_, 25 °C): δ 34.0 (CH_2_-1), 18.9 (CH_2_-2), 200.5 (C-3), 122.6 (CH-4), 165.6 (C-5), 124.3 (CH-6), 134.6 (CH-7), 124.3 (C-8), 44.2 (CH-9), 36.8 (C-10), 25.4 (CH_2_-11), 34.0 (CH_2_-12), 44.0 (C-13), 156.7 (C-14), 35.5 (CH_2_-15), 27.7 (CH_2_-16), 55.6 (CH-17), 18.9 (CH_3_-18), 16.6 (CH_3_-19), 39.3 (CH-20), 21.2 (CH_3_-21), 134.9 (CH-22), 132.2 (CH-23), 42.8 (CH-24), 33.1 (CH-25), 19.6 (CH_3_-26), 19.9 (CH_3_-27), 17.6 (CH_3_-28); (−)ESIMS *m*/*z* = 391.6 [M − H]^−^.

*The acetone adduct of compound **4*** (**4a**): Yellowish brown powder (10 mg; 1.0 mg·L^−1^); UV λ_max_ MeOH (log ε): 331 (4.01), 255 (3.91), 216 (3.81) nm; IR ν_max_ (ATR): 2919, 2850, 2359, 1708, 1608, 1460, 1379, 1206, 836, 720, 668, 534 cm^−1^; ^1^H- and ^13^C-NMR see supp. info. Table 3; (+)-HRESIMS: *m*/*z* found = 421.1258 [M + Na]^+^ and *m*/*z* calcld = 421.1264 [M + Na]^+^.

*The acetone adduct of compound **5*** (**5a**): Yellowish brown powder (12 mg; 1.2 mg·L^−1^); ^1^H- and ^13^C-NMR see supp. info. Table S4; (+)-HRESIMS: *m*/*z* found = 421.1260 [M + Na]^+^ and *m*/*z* calcld= 421.1264 [M + Na]^+^.

*The hydrated form of compound **6*** (**6a**): Yellowish green powder (6.5 mg; 0.6 mgL^−1^); UV λ_max_ MeOH (log ε): 385 (4.91), 242 (4.21) nm; IR ν_max_ (ATR): 3380, 2920, 2853, 2458, 1740, 1717, 1610, 1465, 1373, 1300, 1195, 1065, 1030, 970, 862 cm^−1^; ^1^H- and ^13^C-NMR see supp. info. Table 5; (+)-HRESIMS: *m*/*z* found = 396.1057 [M + Na]^+^ and *m*/*z* calcld = 396.1059 [M + Na]^+^.

### 3.4. Biological Activity

Cell Lines. The human cancer cell lines K562 (chronic myelogenous leukemia), U266 (myeloma), SKM1 (myelodysplastic symdrom), and Kasumi (acute myeloid leukemia) were provided by ATCC and were grown at 37 °C under 10% CO_2_ in RPMI 1640 medium (Gibco BRL, Paisley, UK) supplemented with 10% FCS (Gibco BRL, Paisley, UK) completed with 50 units/mL penicillin, 50 mg/mL streptomycin, and 1 mM sodium pyruvate.

Measurement of Cell Metabolism (XTT). Cells (10 × 10^4^/mL) were incubated with **1** or **2** for the times indicated. 50 µL of XTT kit (sodium 39-[1-(phenylaminocarbonyl)-3,4-tetrazolium]-bis(4-methoxy-6-nitro)benzene sulfonic acid hydrate) was added to each well, which contain 100 µL of medium. Absorbance of the formazan dye produced by metabolically active cells was measured at 490 nm. Each assay was performed in quadruplicate.

### 3.5. Molecular Modeling

All the modeling studies were carried out on a laptop PC, Intel^©^ Core™ i7-3630QM CPU @ 2.40 GHz, RAM 8 GB operating under Windows 7 professional. It consists of several steps. First, the 3D crystal structures of ERα-LBD with PDB code 1A52 [15] were downloaded from Brookhaven Protein Data loaded to Molegro Virtual Docker (MVD 2013.6.0.0 [Win32], CLC Bio Company, Aarhus, Denmark) program fully functional free trial version with time limiting license (Molegro Virtual Docker (MVD 2013.6.0). ChemBio3D Ultra 10 [16] was used to draw the 3D structures of different ligands. Ligands were further pre-optimized using a free version of Marvinsketch 4.1.13 from Chemaxon Ltd (Marvinsketch, version 6.1.0, ChemAxon company cheminformatics technology products services, Budapest, Hungary, 2013, http://www.chemaxon.com) with MM force field and saved in Tripos mol2 file format. MolDock score functions were used with a 0.3 Å grid resolution. Prior to the calculations of the subject compounds, the MVD software was benchmarked docking the Estrogen.

## 4. Conclusions

Marine fungi are promising inspiring sources for new chemical skeletons. This work introduced new and promising alkaloidal phenalenone derivatives from the marine-derived endophytic fungus *Coniothyrium cereale*. The unusual heterodimer compounds **1** and **2** are composed of a sterol portion condensed to phenalenone derivatives. Compound **6** contains unprecedented imine functionality between two carbonyl groups to form an oxepane-imine ring.

## Supplementary Materials

Supplementary materials can be accessed at: www.mdpi.com/1420-3049/21/2/178. 1D- and 2D-NMR data for compounds **1**, **2**, and **6**, and the NOESY correlations of compound **1**, 1D-NMR data for compounds **3**–**5**, for online publication.
