# Supplementary material for: Unusual Nitrogenous Phenalenone Derivatives from the Marine-Derived Fungus Coniothyrium cereale
Source: Molecules. 2016 Feb 1;21(2):178. doi: 10.3390/molecules21020178 (PMC6273853; doi:10.3390/molecules21020178)
Supplement: Supplementary file 1 [file molecules-21-00178-s001.docx]

Unusual Nitrogenous Phenalenone Derivatives from the Marine-Derived Fungus *Coniothyrium cereale*

Mahmoud Fahmi Elsebai, Hazem A. Ghabbour and Mohamed Mehiri


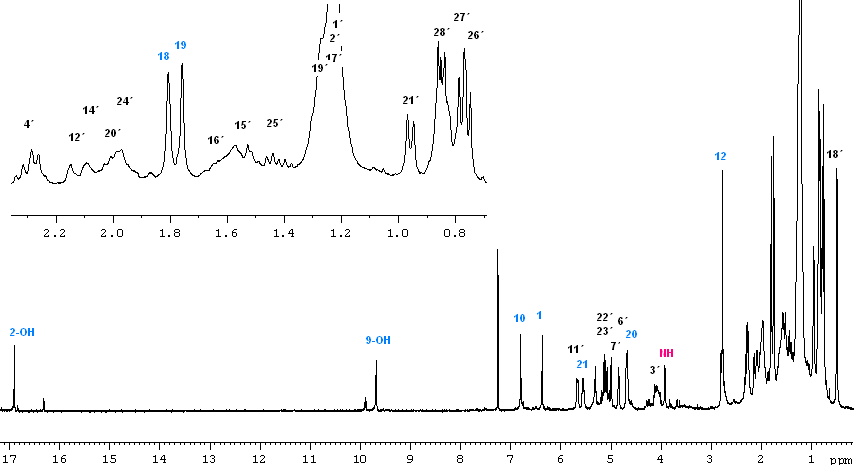


**Figure S1.** ^1^H-NMR spectrum (300 MHz, CDCl_3_) of conio-azasterol (**1**).


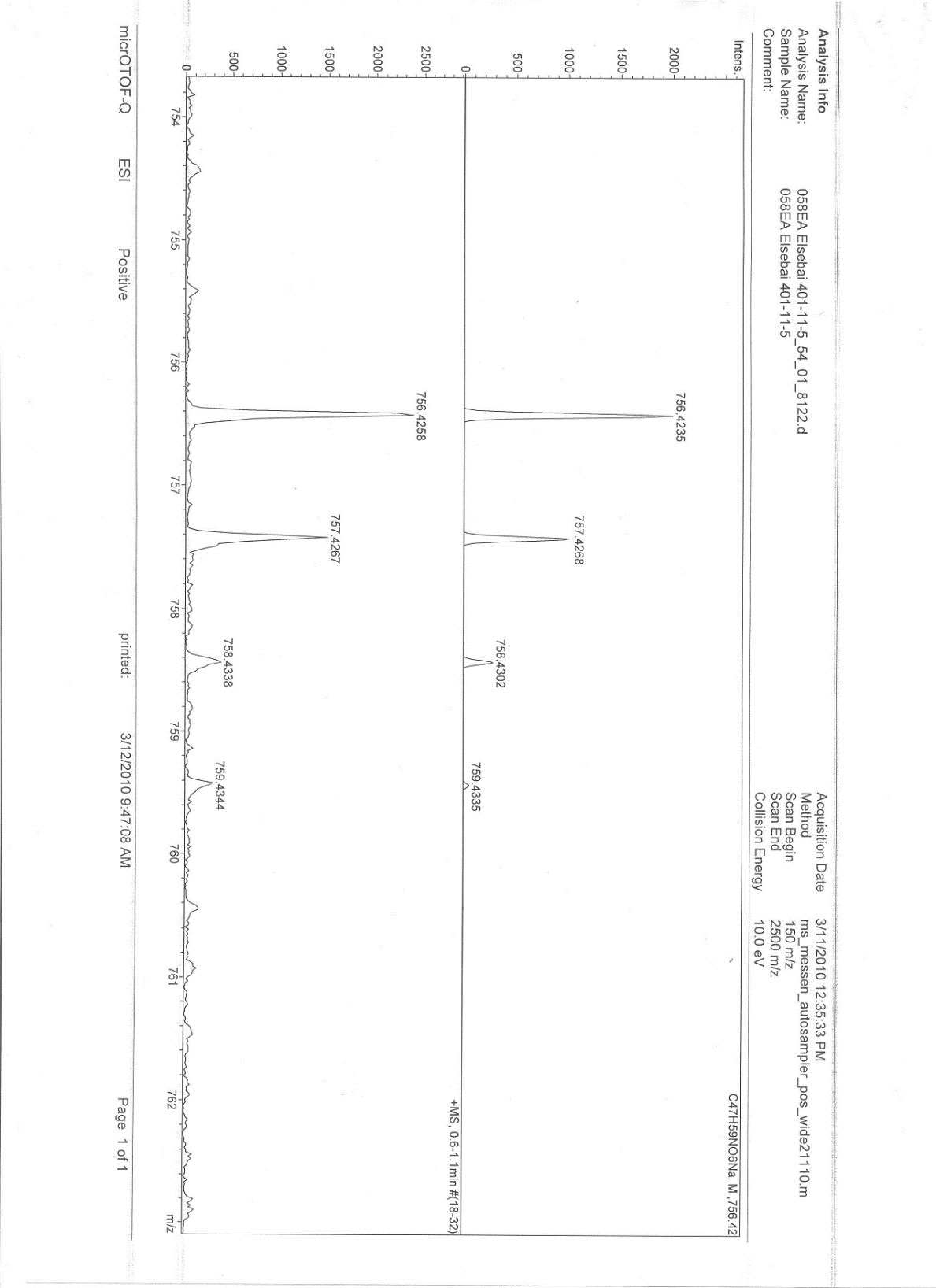


**Figure S2.** (+)-HRESIMS of conio-azasterol (**1**).


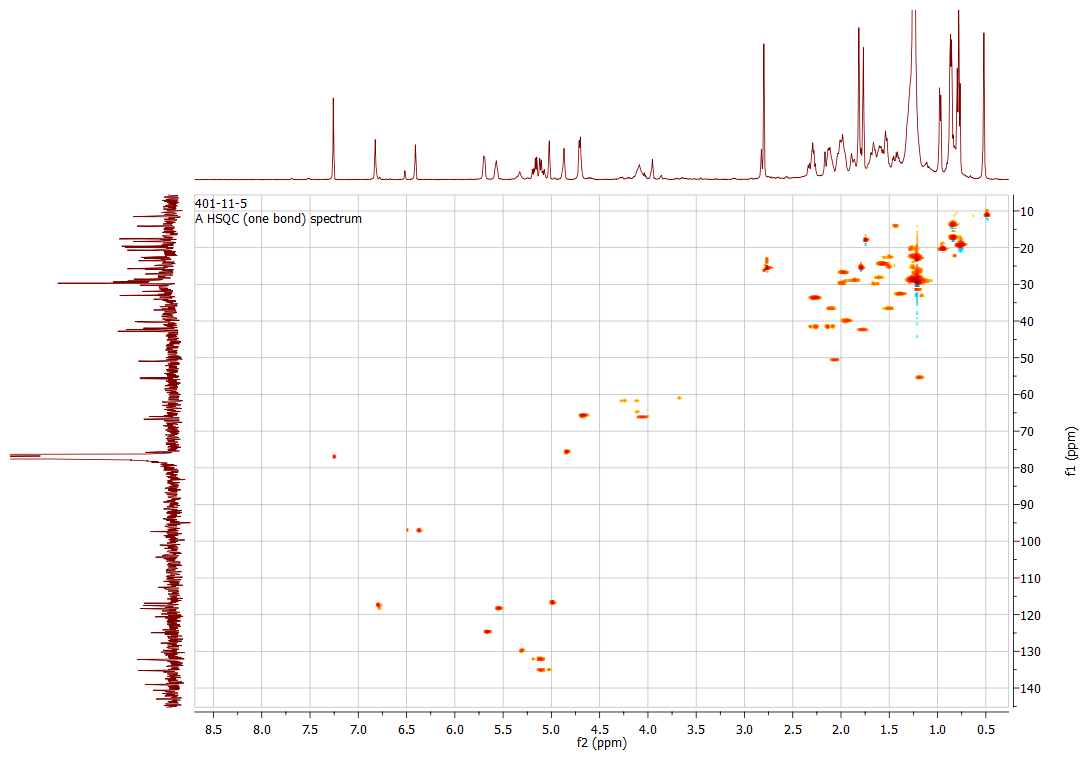


**Figure S3.** HSQC spectrum of conio-azasterol (**1**).


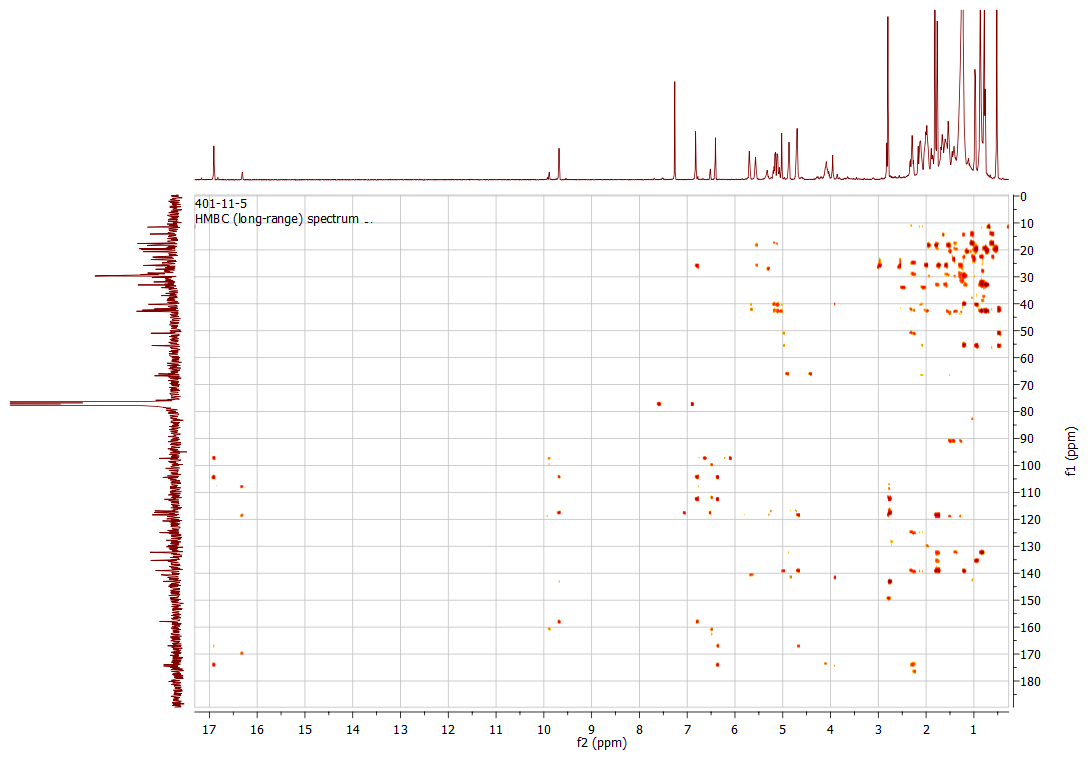


**Figure S4.** HMBC spectrum of conio-azasterol (**1**).


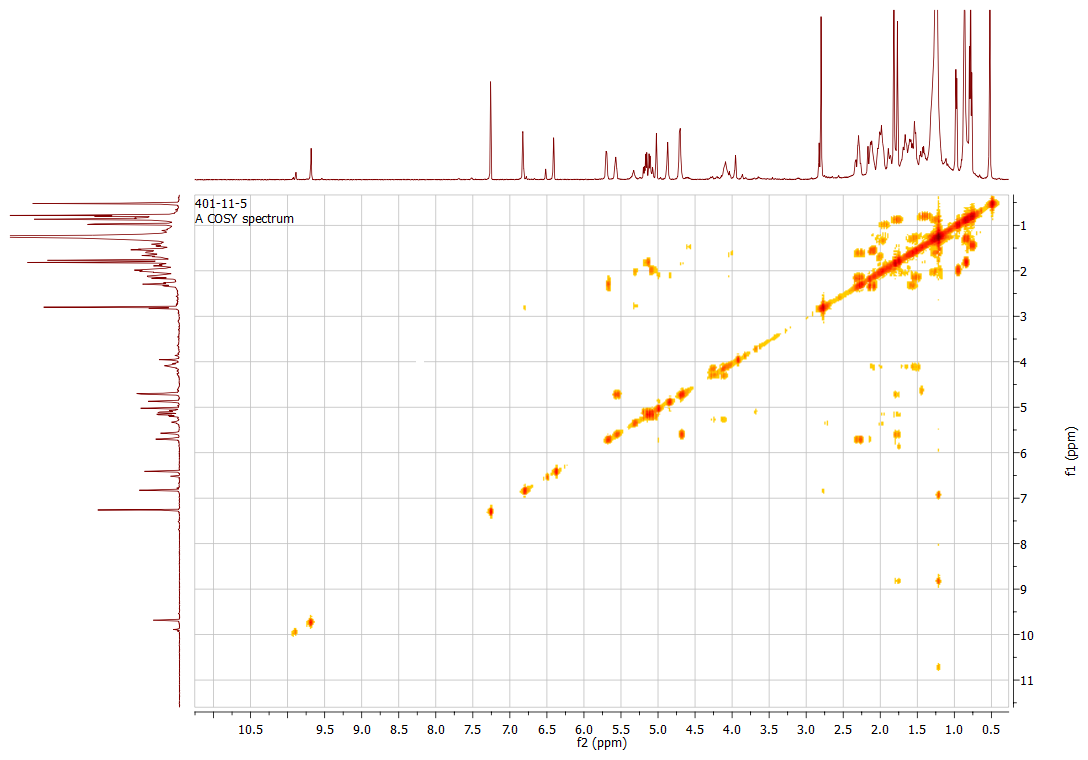


**Figure S5.** COSY spectrum of conio-azasterol (**1**).


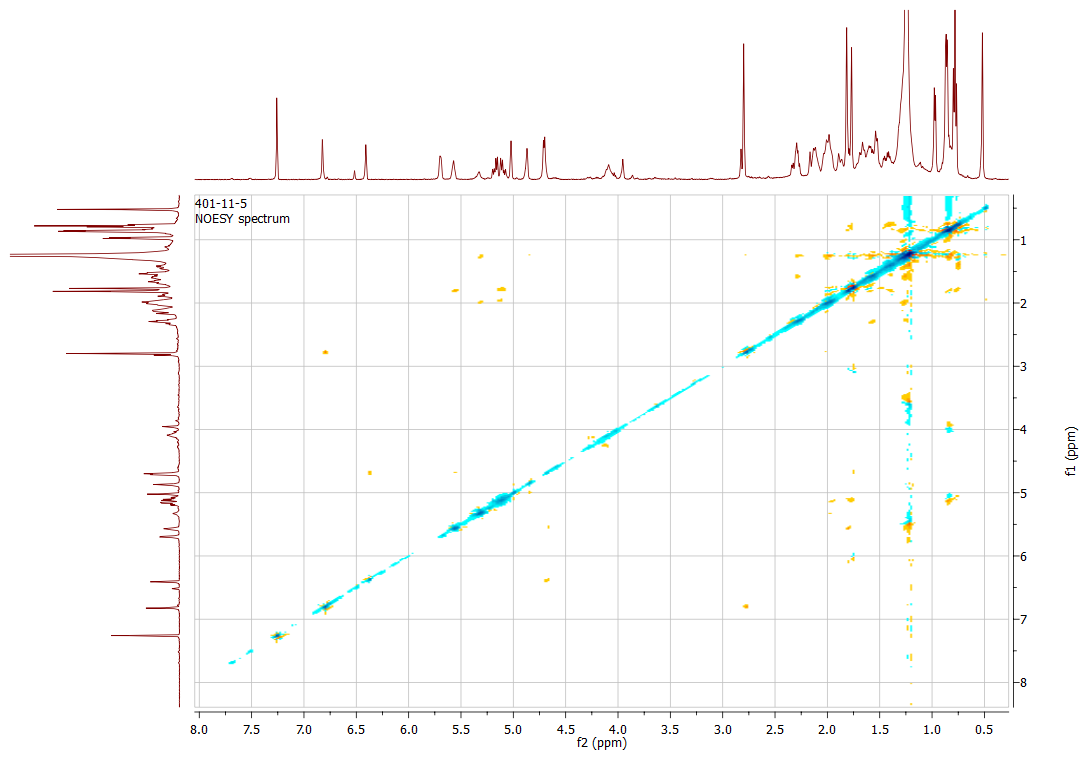


**Figure S6.** NOESY spect0rum of conio-azasterol (**1**).

|  |
| --- |
| 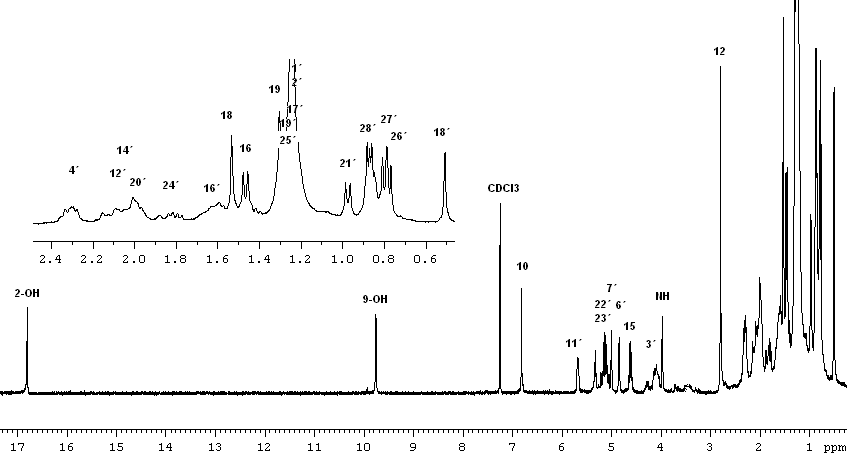 |

**Figure S7.** ^1^H-NMR spectrum (300 MHz, CDCl_3_) of *S*-dehydroazasirosterol (**2**).


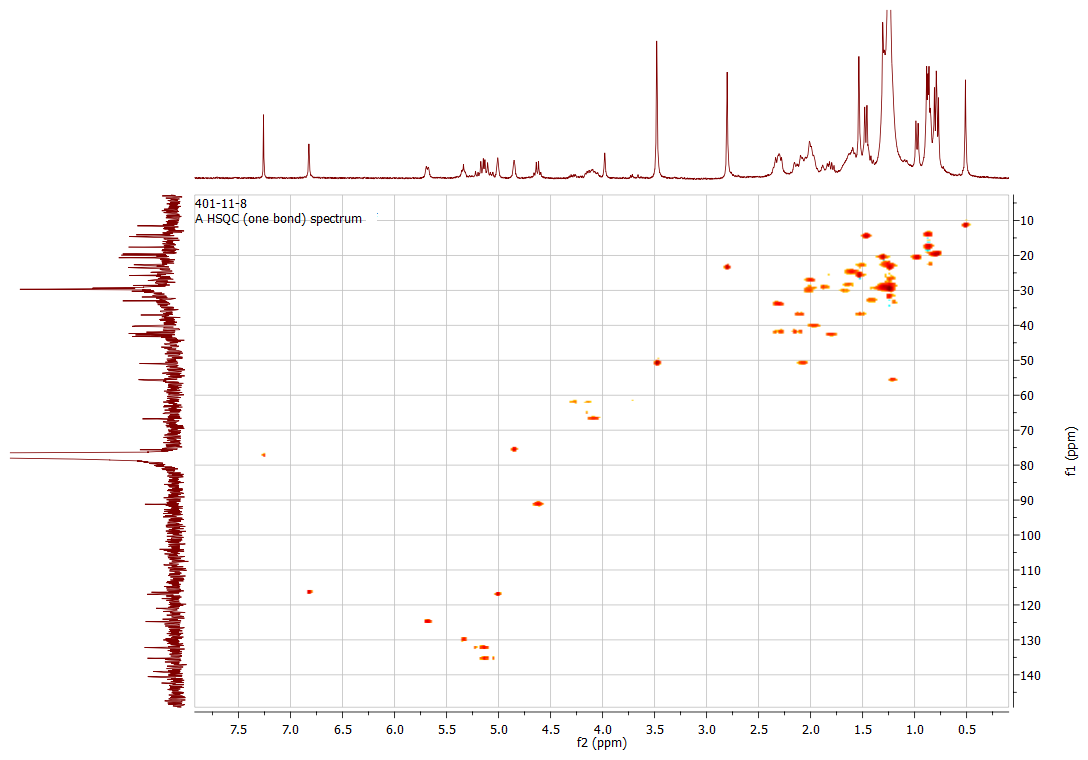


**Figure S8.** HSQC spectrum of *S*-dehydroazasirosterol (**2**).


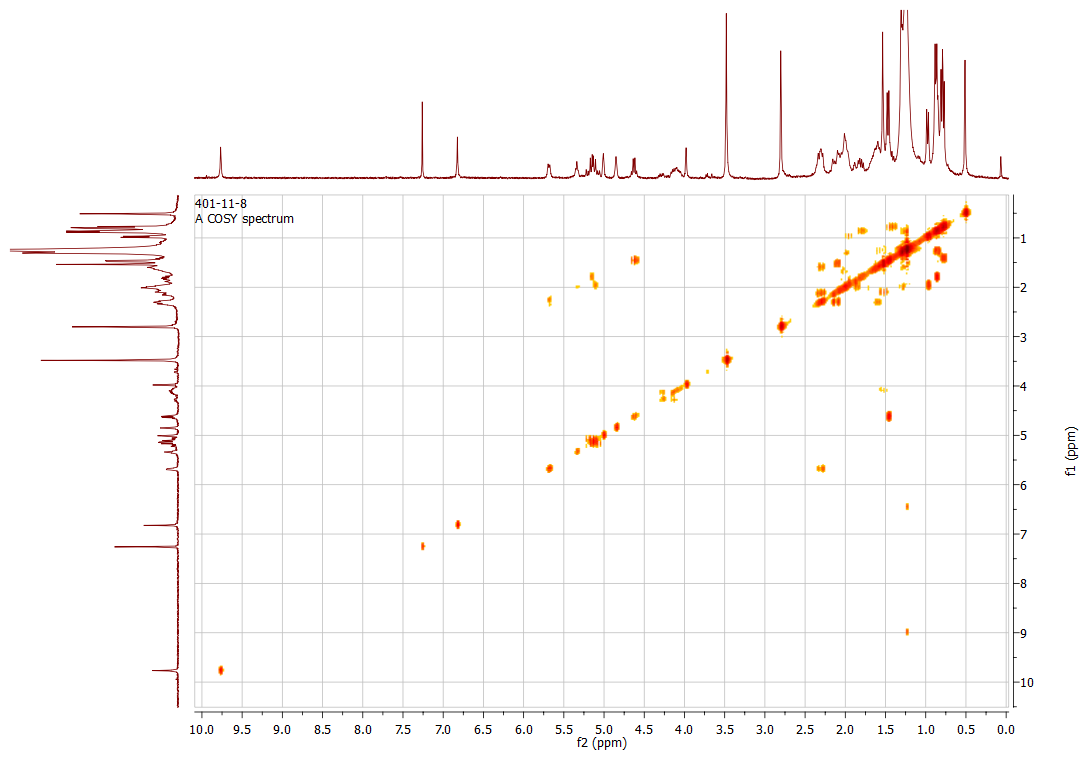


**Figure S9.** COSY spectrum of *S*-dehydroazasirosterol (**2**).


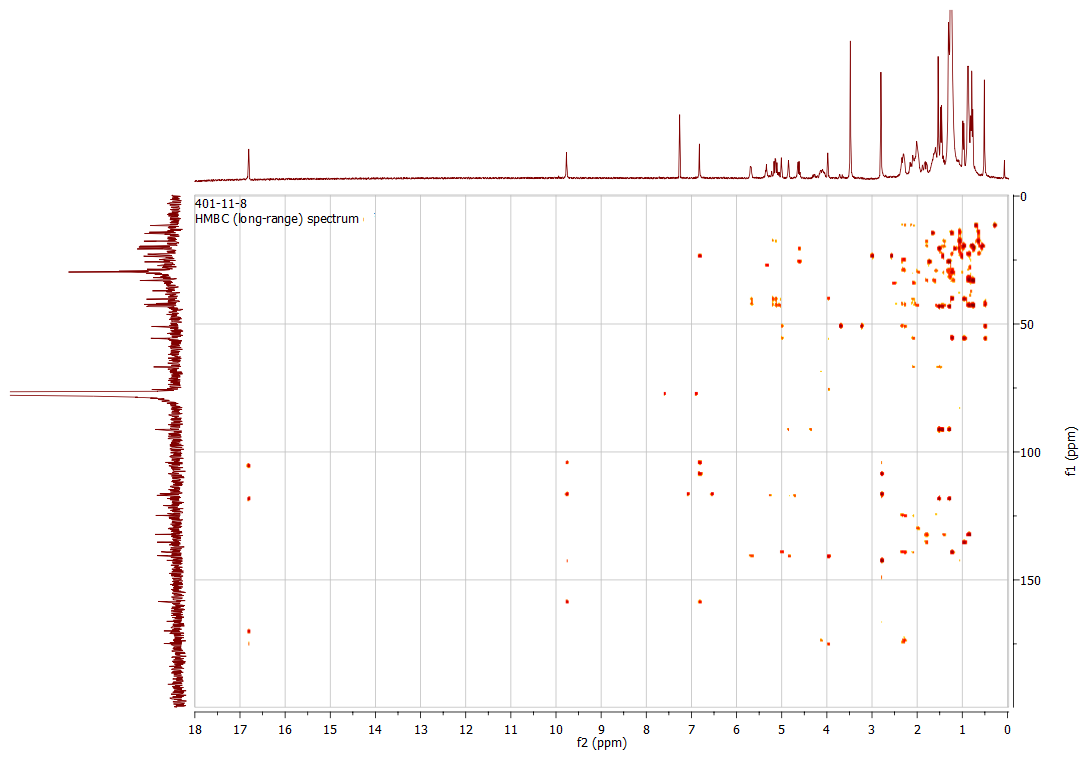


**Figure S10.** HMBC spectrum of *S*-dehydroazasirosterol (**2**).


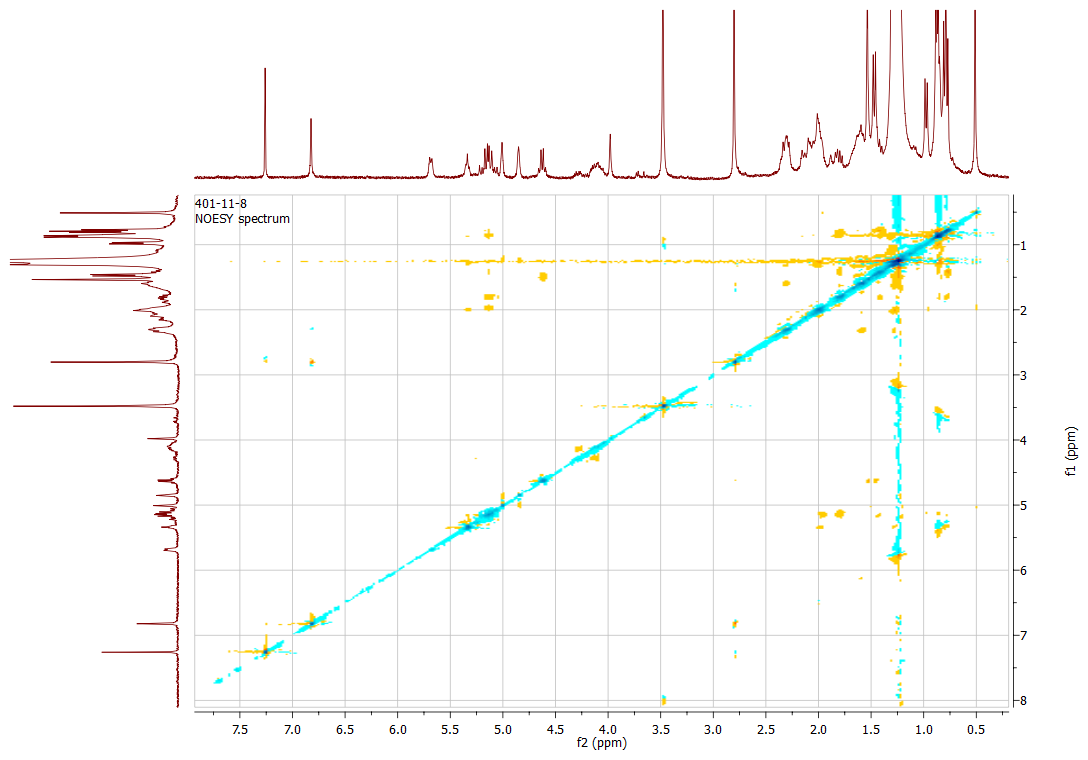


**Figure S11.** NOESY spectrum of *S*-dehydroazasirosterol (**2**).

**Figure S12.** Significant ^1^H-^1^H 2D NOESY correlations of *S*-dehydroazasirosterol (**2**).


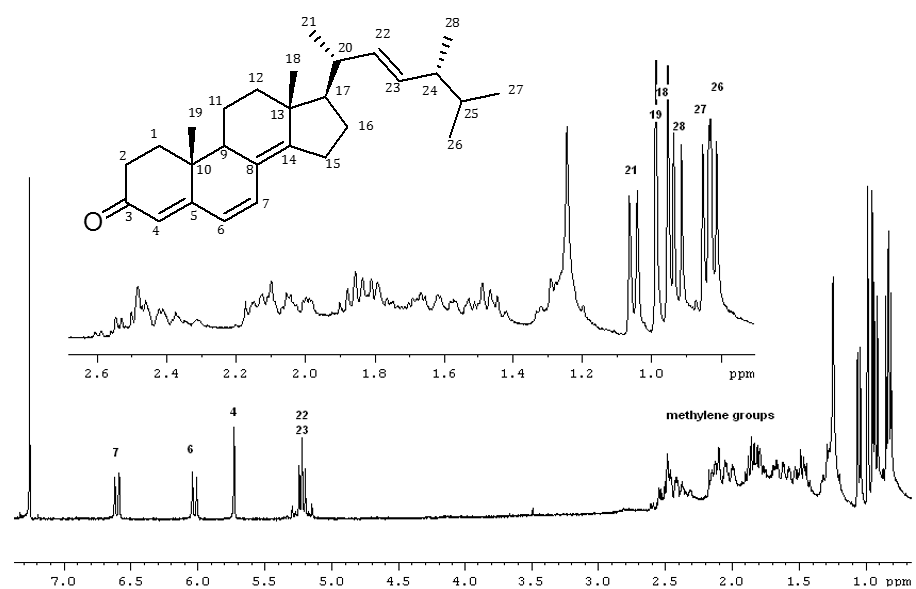


**Figure S13.** ^1^H-NMR spectrum (300 MHz, CDCl_3_) of compound **3**.


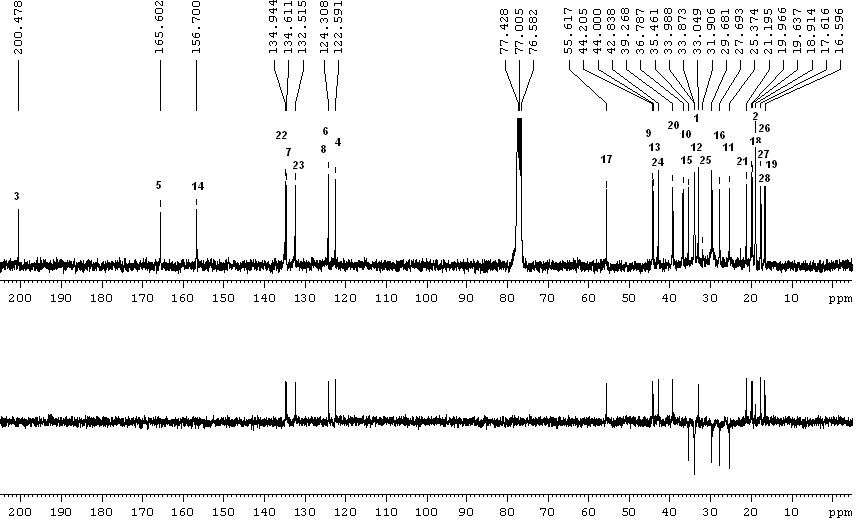


**Figure S14.** ^13^C-NMR (75 MHz, CDCl_3_) and DEPT (135) spectra of compound **3**.


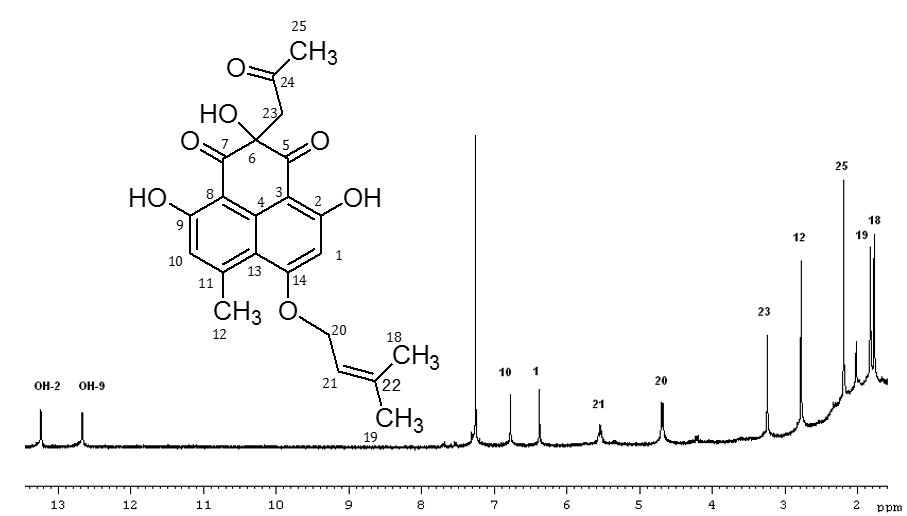


**Figure S15.** ^1^H-NMR spectrum (300 MHz, CDCl_3_) of **4a**.


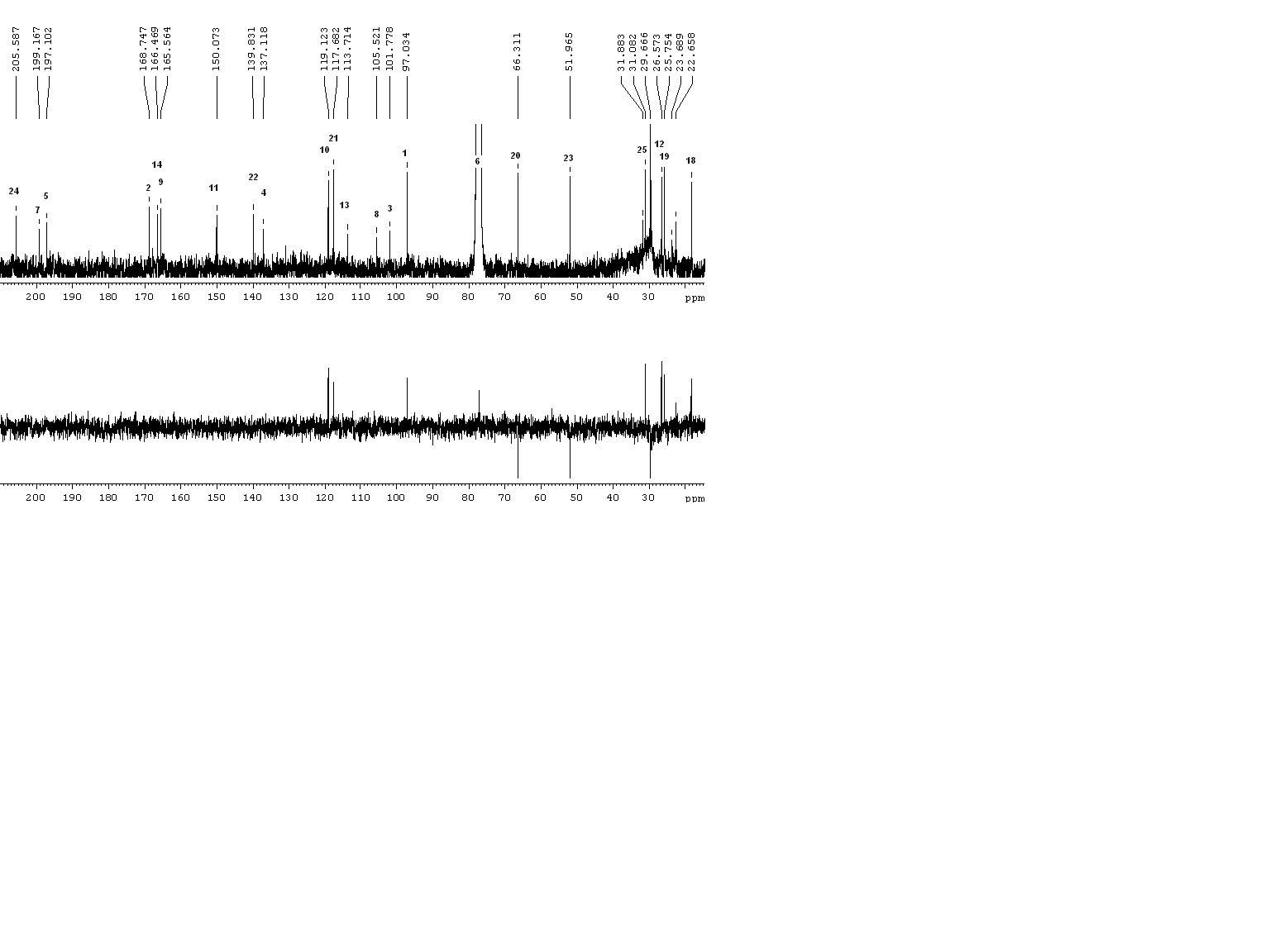


**Figure S16.** ^13^C-NMR (75 MHz, CDCl_3_) and DEPT (135) spectra of **4a**.


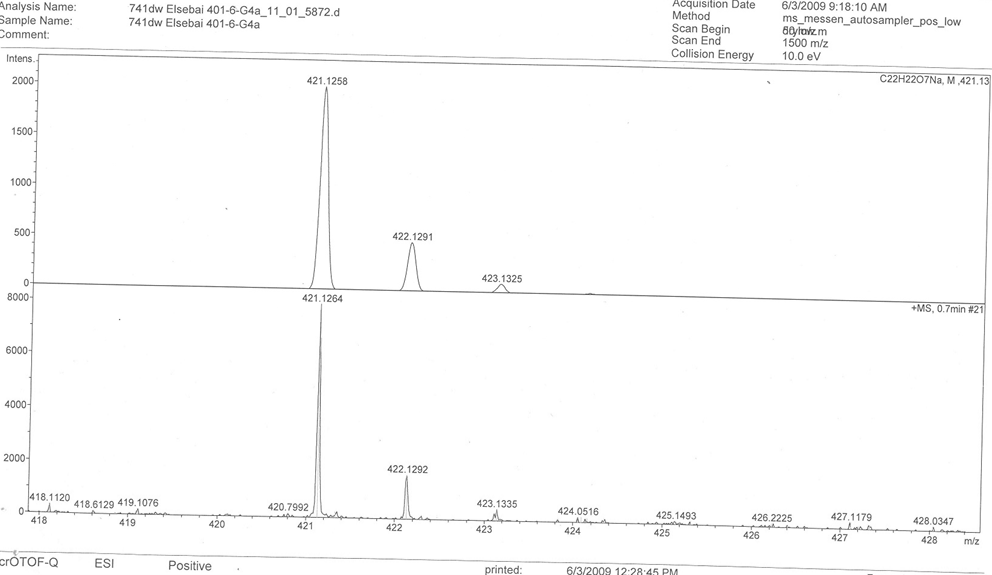


**Figure S17.** (+)-HRESIMS of **4a**.


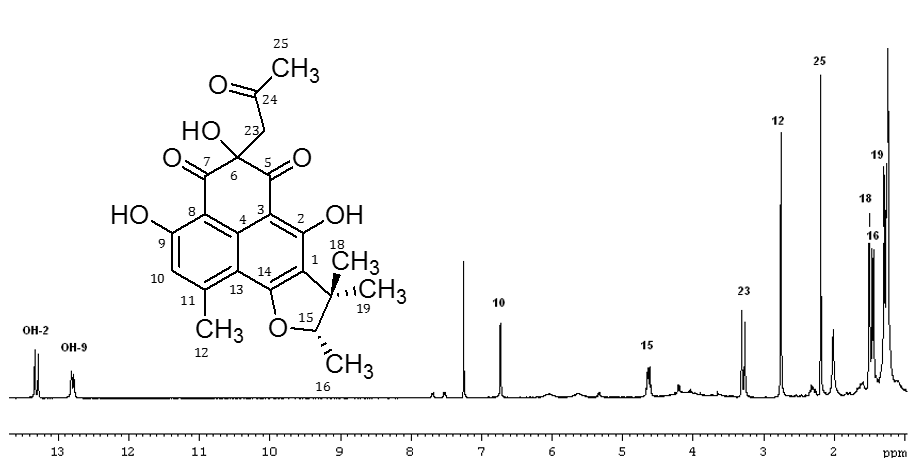


**Figure S18.** ^1^H-NMR spectrum (300 MHz, CDCl_3_) of **5a**.


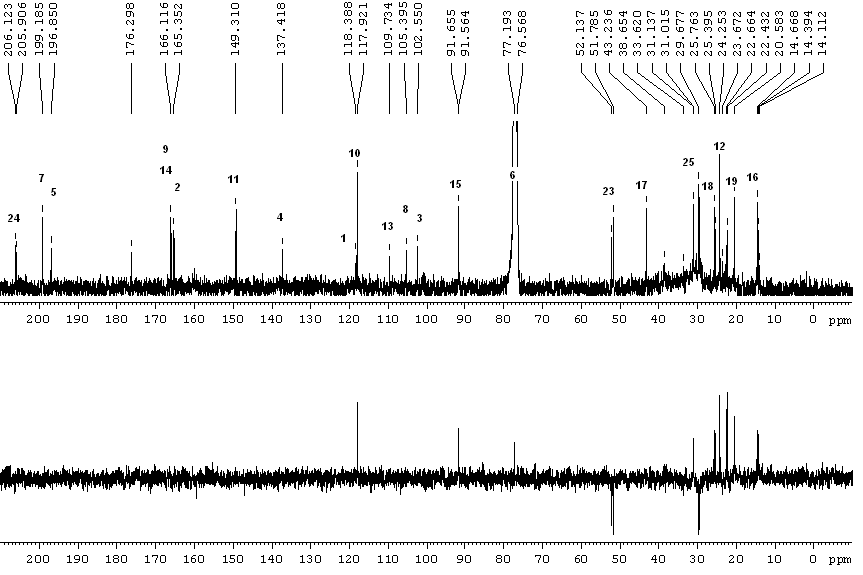


Figure S19. ^13^C-NMR (75 MHz, CDCl_3_) and DEPT (135) spectra of **5a**.


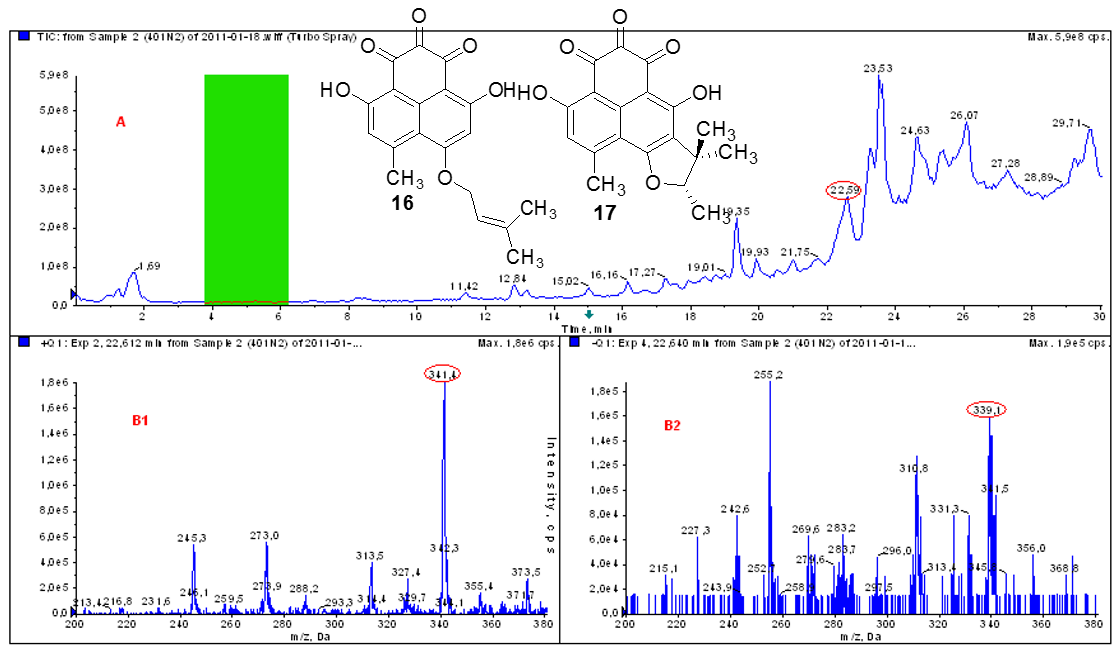


**Figure S20.** LC/MS analysis of a new extract of *Coniothyrium cereale* under nitrogen. The chromatogram of LC/MS showed the molecular masses of the triketone compounds 4 and 5 at *m*/*z* 341 Da [M + H]^+^ and m/z 339 Da [M – H]^+^. This new extraction has been done under nitrogen and without using acetone. Therefore, it showed the genuine triketone compounds (4 and 5) and not their acetone adducts (4a and 5a).


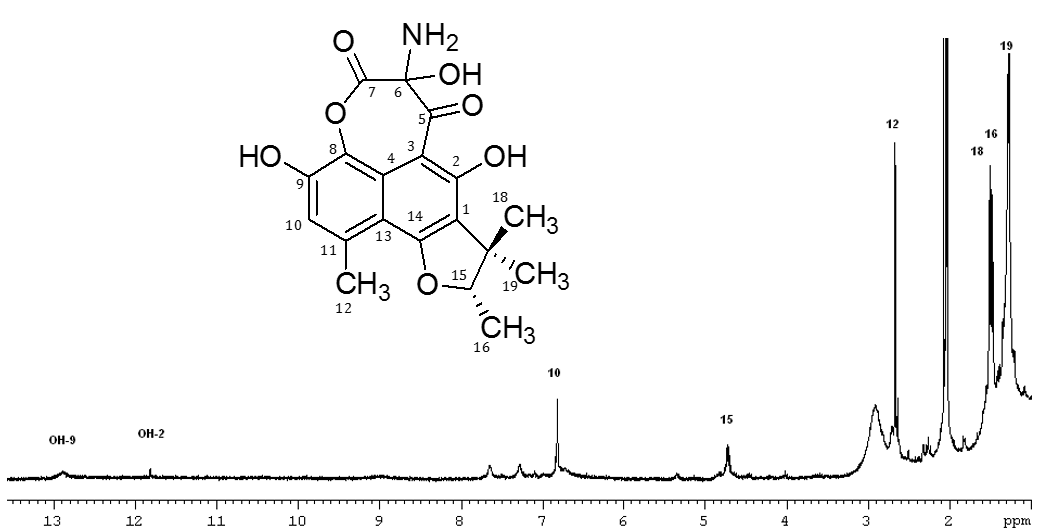


Figure S21. ^1^H-NMR spectrum (300 MHz, CD_3_COCD_3_) of **6a**.


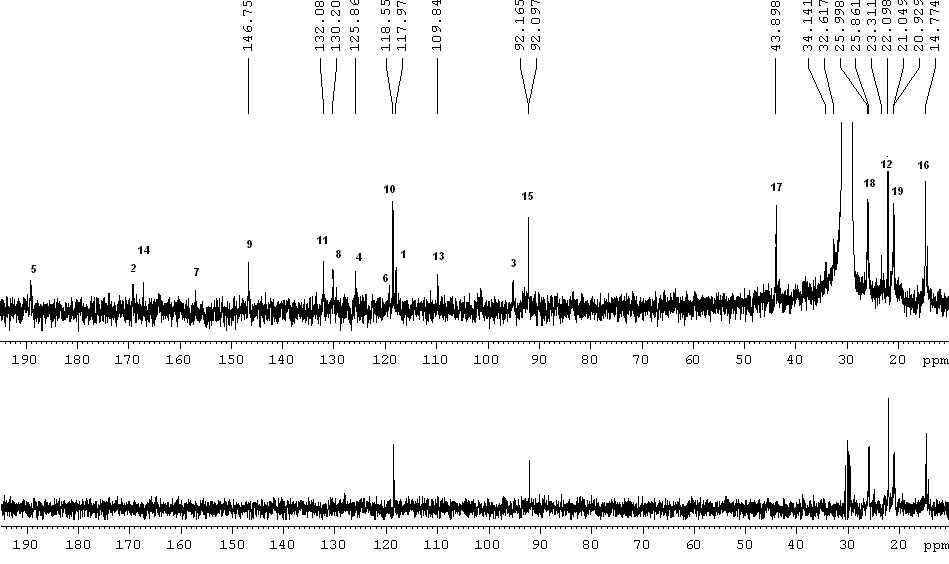


Figure S22. ^13^C-NMR (75 MHz, CD_3_COCD_3_) and DEPT (135) spectra of **6a**.


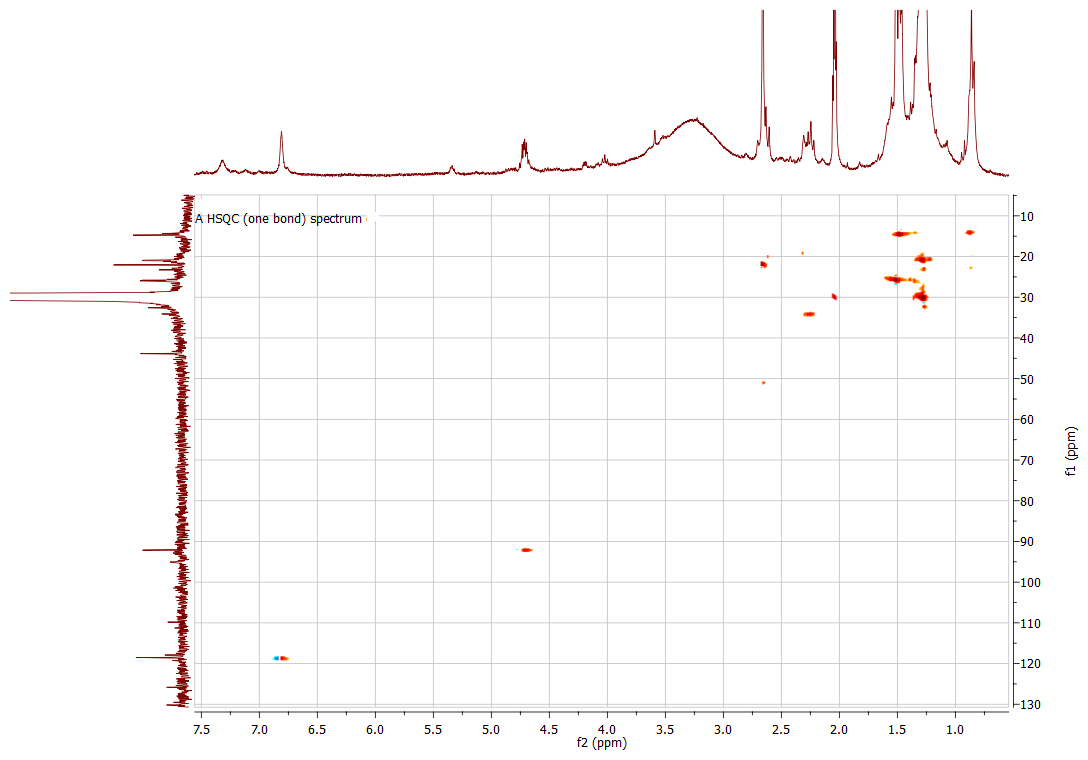


Figure S23. HSQC spectrum of compound **6a**.


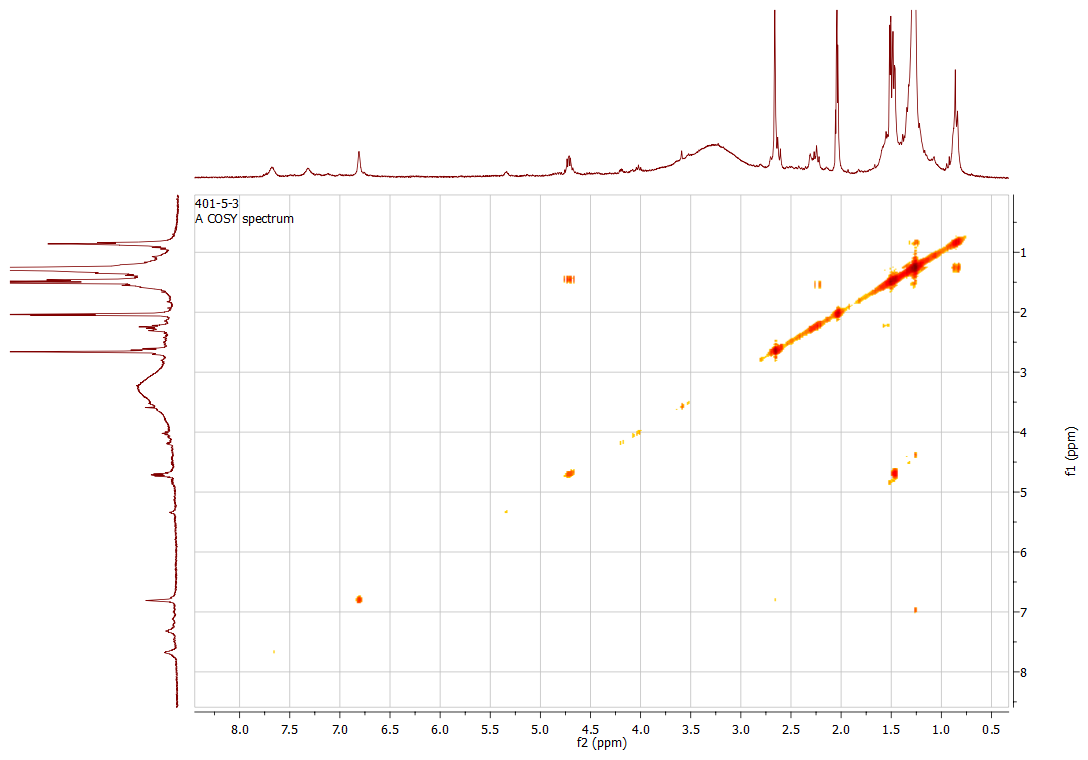


**Figure S24.** COSY spectrum of compound **6a**.


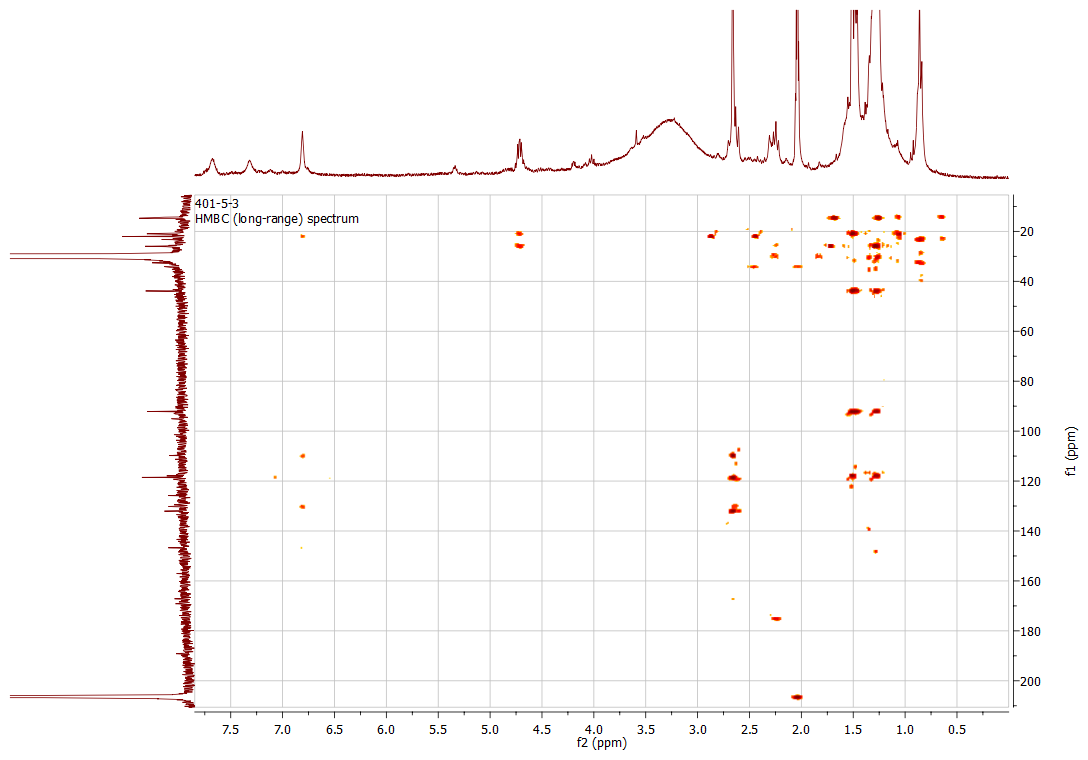


**Figure S25.** HMBC spectrum of compound **6a**.
